# *Hericium erinaceus* Promotes Anti-Inflammatory Effects and Regulation of Metabolites in an Animal Model of Cerebellar Ataxia

**DOI:** 10.3390/ijms24076089

**Published:** 2023-03-23

**Authors:** Sze Chun Chau, Pit Shan Chong, Hongkai Jin, Ka Chun Tsui, Sharafuddin Khairuddin, Anna Chung Kwan Tse, Sze Yuen Lew, George Lim Tipoe, Chi Wai Lee, Man-Lung Fung, Kah Hui Wong, Lee Wei Lim

**Affiliations:** 1 School of Biomedical Sciences, Li Ka Shing Faculty of Medicine, The University of Hong Kong, Hong Kong, China; 2 Department of Anatomy, Faculty of Medicine, Universiti Malaya, Kuala Lumpur 50603, Malaysia

**Keywords:** *Hericium erinaceus*, cerebellar ataxia, incoordination, brain inflammation, neuroprotective agents, neurotransmission

## Abstract

Cerebellar ataxia is a neurodegenerative disorder with no definitive treatment. Although previous study demonstrated the neuroprotective effects of *Hericium erinaceus* (*H.E.*), the mechanisms of *H.E*. treatment on the neuroinflammatory response, neurotransmission, and related metabolites remain largely unknown. We demonstrated that 3-AP rats treated with 25 mg/kg *H.E.* extracts had improved motor coordination and balance in the accelerated rotarod and rod tests. We showed that the *H.E*. treatment upregulated the expression of *Tgfb1, Tgfb2,* and *Smad3* genes to levels comparable to those in the non-3-AP control group. Interestingly, we also observed a significant correlation between *Tgfb2* gene expression and rod test performance in the 3-AP saline group, but not in the non-3-AP control or *H.E*.+3-AP groups, indicating a relationship between *Tgfb2* gene expression and motor balance in the 3-AP rat model. Additionally, we also found that the *H.E*. treatment increased mitochondrial COX-IV protein expression and normalized dopamine-serotonin neurotransmission and metabolite levels in the cerebellum of the *H.E*.+3-AP group compared to the 3-AP saline group. In conclusion, our findings suggest that the *H.E*. treatment improved motor function in the 3-AP rat model, which was potentially mediated through neuroprotective mechanisms involving TGFB2-Smad3 signaling via normalization of neurotransmission and metabolic pathways.

## 1. Introduction

Cerebellar ataxia is a progressive motor disorder characterized by dyssynergia, dysmetria, balance impairment, and gait instability [1,2]. The etiology of this disorder can be sporadic or hereditary [1,3], with the majority of cases showing neurodegeneration in the cerebellum leading ataxia [4,5]. The ataxic motor dysfunctions are associated with alterations in cerebellar circuit connectivity and Purkinje cell functions [6,7,8,9]. Lesions in Purkinje cells and connected region have been shown to induce motor and balance impairments in ataxic-like animal models [10,11]. Currently, there are no effective treatments for cerebellar ataxia due to its complex pathophysiology [12]. Existing physical therapy or training offer only limited therapeutic effects against motor and balance deterioration. Therefore, an effective treatment is needed to improve or alleviate the ataxic symptoms [12].

A growing number of studies have shown that medicinal mushrooms can have beneficial effects in neurodegenerative diseases [13,14,15,16,17]. These mushrooms contain various bioactive compounds with anti-inflammatory and antioxidative properties that can target multiple therapeutic pathways [16,18]. Although studies have shown that extracts of medicinal mushroom can improve motor function in ataxia models, there is a lack of mechanistic study on their therapeutic effects [19,20]. *Hericium erinaceus* (*H.E.*) or lion’s mane mushroom is a medicinal mushroom that has long been used as a traditional Chinese medicine. It is well known for its diverse beneficial effects on the brain, including neurotrophic [21,22], antiaging [17,23], and antioxidative [24,25] activities. Recent studies have reported that *H.E*. has therapeutic potential against neurological disorders including Alzheimer’s disease [17,26,27], Parkinson’s disease [18,28,29], frailty [23,30], depression [31,32], and obesity-induced neurodegeneration [33]. Furthermore, *H.E.* has also been suggested to have neuroprotective activity against cerebellar ataxia [19,24]. Our recent study showed that *H.E.* had neuroprotective effects in a rat model of neurotoxin 3-acetylpyridine (3-AP)-induced cerebellar ataxia [19]. We observed that *H.E.* treatment in 3-AP rats normalized the altered Purkinje cell morphology and enhanced the expression of neuroplasticity-related proteins including pERK1/2, pCREB, and postsynaptic density protein 95 (PSD95) [19]. Although these studies show that *H.E.* has neuroplasticity-related activities, the mechanisms of the anti-inflammatory and neurotransmission effects of *H.E.* in cerebellar ataxia remain obscure. In this study, we investigated the effects of *H.E.* on genes related to pro- and anti-inflammatory pathways, proteins related to mitochondria and neurotransmission, and metabolites related to glycolysis, tricarboxylic acid cycle, sugars, and amino acids.

## 2. Results

### 2.1. H.E. Treatment Rescues Body Weight Loss and Motor Deficits

In the assessment of baseline body weight, one-way ANOVA (F_(2,17)_ = 1.622, *p =* n.s.) showed no remarkable differences among groups (Supplementary Figure S7A) on the day before 3-AP administration. On day 2 and on weeks 1, 2, and 3 after 3-AP injection, we found a significant reduction in body weight in the 3-AP group compared to the control group (all *p* < 0.001). At 1, 2, and 3 weeks, the body weight of *H.E.*+3-AP animals increased compared to 3-AP animals (all *p* < 0.041). Accelerated rotarod and rod tests were conducted to investigate motor coordination and balance deficits. A non-parametric Kruskal–Wallis test showed a significantly increased percentage deficit in the accelerated rotarod test in 3-AP animals, indicating impaired performance compared to control animals (all *p* < 0.001; Supplementary Figure S7C). After *H.E.* treatment, there was a remarkable improvement in the percentage deficit observed at 2 weeks (*p =* 0.039) and a marginal decrease in the percentage deficit at 3 weeks (*p =* 0.053) in the *H.E.*+3-AP group compared to the 3-AP group. In the rod test, we found that motor balance was impaired in the 3-AP group compared to the control group at 2 and 3 weeks (all *p* < 0.022; Supplementary Figure S7D). Interestingly, *H.E.* treatment significantly restored the balance and motor deficits to levels comparable to those of control animals at 2 and 3 weeks (all *p <* 0.039).

### 2.2. H.E. Treatment Enhances Anti-Inflammatory and Tgfb-Smad3 Genes

**Figure 1:**
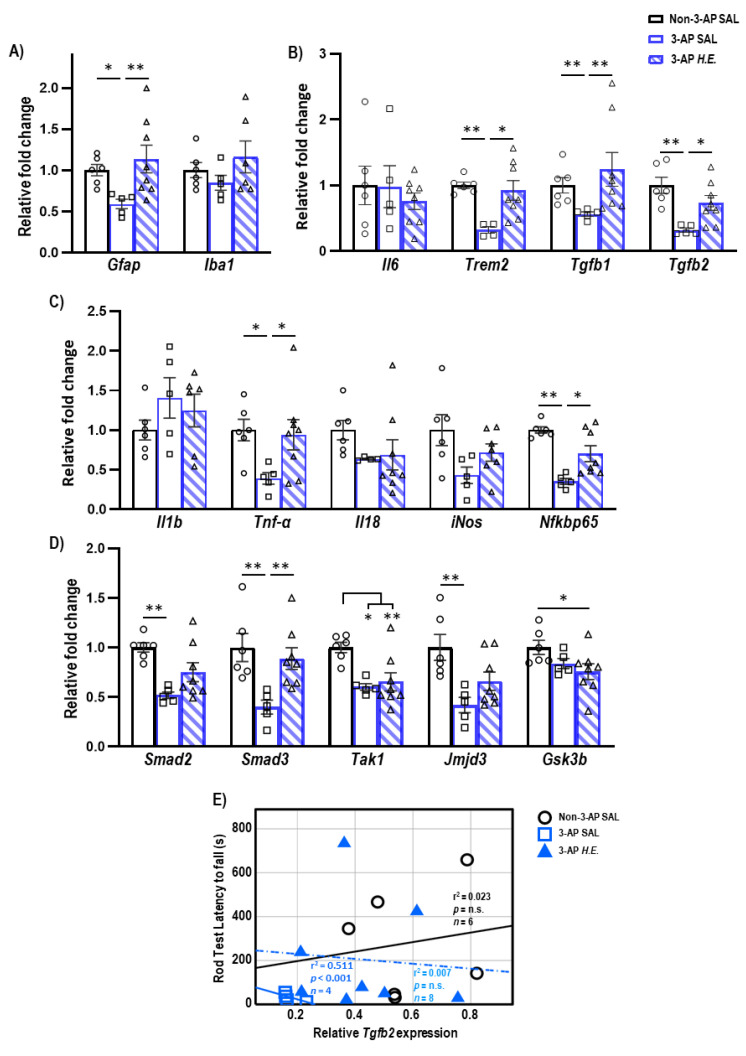
Effects of 3-AP and *H.E*. treatment on the relative expression of inflammatory genes in the cerebellum. (**A**) Relative expression of glial-related genes: *Gfap* and *Iba1*. (**B**) Relative expression of anti-inflammatory genes: *Il6*, *Trem2*, *Tgfb1,* and *Tgfb2*. (**C**) Relative expression of pro-inflammatory genes: *Il1b*, *Tnf*-α, *Il18*, *iNos*, and *Nfkbp65*. (**D**) Relative expression of TGF-β signaling-related genes: *Smad2*, *Smad3*, *Tak1*, *Jmjd3,* and *Gsk3b*. (**E**) Correlational analysis between *Tgfb2* genes and latency to fall in the rod test. Indicators: * *p*-values ≤ 0.05, ** *p*-values ≤ 0.01, ◯ Non-3-AP SAL group, ☐ 3-AP SAL group, △ 3-AP *H.E.* group.

To investigate the neuroprotective effects of *H.E.* in the 3-AP model, qPCR was carried out to analyze changes in glial-(Figure 1A), inflammatory-(Figure 1B,C), and TGF-β signaling-related genes (Figure 1D). We found a significant reduction in the expression of *Gfap* (*p =* 0.012) in 3-AP animals compared to controls. The *H.E.* treatment remarkably increased the mRNA level of *Gfap* (*p =* 0.006) to a level comparable to that of the controls (Figure 1A). No remarkable changes were found for *Iba1* when comparing all groups (*p =* n.s.). In addition, we found that the 3-AP group had significantly reduced expression of *Trem2* (*p =* 0.004), *Tgfb1* (*p =* 0.005), and *Tgfb2* (*p =* 0.001) compared to the controls (Figure 1C). Interestingly, *H.E.* treatment normalized the expression of these genes to levels comparable to those of the control (all *p* < 0.04; Figure 1B), indicating that *H.E.* has anti-inflammatory effects in the 3-AP model. No significant difference was found in the expression of *Il6* among all groups (*p =* n.s.). We also found that 3-AP animals had remarkably decreased expression of *Tnf*-α (*p =* 0.015) and *Nfkbp65* (*p =* 0.001) compared to the controls (Figure 1C). Notably, the *H.E.*+3-AP group showed restored expressions of *Tnf*-α (*p =* 0.04) and *Nfkbp65* (*p =* 0.02) compared to the 3-AP group. There were no remarkable differences in the gene expression of *Il1b*, *Il18*, or *iNos* among all groups (all *p =* n.s.). Moreover, the 3-AP group had a decreased expression of *Smad2*, *Smad3*, *Tak1,* and *jmjd3* compared to the control group (all *p* < 0.027; Figure 1D). The *H.E.*+3-AP group showed upregulated *Smad3* levels (*p =* 0.006) but no significant changes in *Smad2* and *jmjd3* compared to the 3-AP group (all *p =* n.s.). In the correlational analysis, we found that the rod test performance was negatively correlated with the expression of *Tgfb2* in the 3-AP group (r^2^ = 0.511, *p* < 0.001; Figure 1E), indicating a relationship between *Tgfb2* and motor balance impairment in the 3-AP model. No remarkable correlation was found between *Tgfb2* expression and percentage deficit in the rotarod test when comparing all groups.

### 2.3. H.E. Enhances COX-IV and pAkt/Akt Ratio

**Figure 2:**
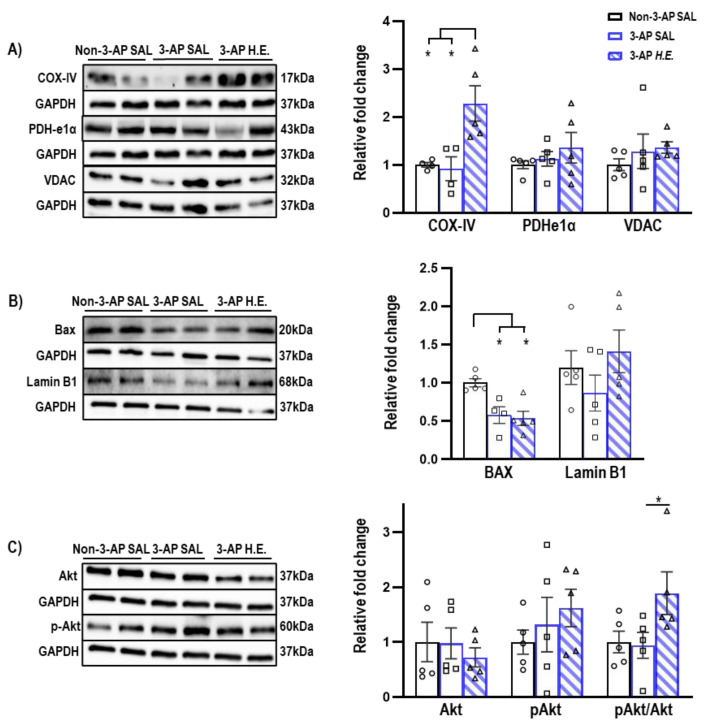
Effects of 3-AP and *H.E*. treatment on relative protein expressions in the cerebellum. (**A**) Relative expressions of mitochondrial-related proteins: COX-IV, PDHe1α, and VDAC. (**B**) Relative expressions of apoptosis-related proteins: BAX, Lamin B1, and (**C**) pAKT/AKT. Indicators: * *p*-values ≤ 0.05, ◯ Non-3-AP SAL group, ☐ 3-AP SAL group, △ 3-AP *H.E.* group.

Proteins related to mitochondrial apoptosis were analyzed to investigate the effects of *H.E.* on apoptotic functions. A non-parametric Kruskal–Wallis test showed that the *H.E.* treatment significantly increased COX-IV protein expression compared to both 3-AP and control animals (all *p* < 0.013; Figure 2A). There was also significantly reduced BAX expression compared to the control group (all *p* < 0.021; Figure 2B). There were no significant differences in the protein expression of PDHe1α, VDAC, or Lamin B1 when comparing all groups (all *p =* n.s.; Figure 2A,B). Considering that a previous study reported that Akt induces the phosphorylation of a mitochondrial ATP synthase beta-subunit [34], we found that the *H.E.* treatment significantly increased the pAkt/Akt ratio in 3-AP animals compared to the control group (*p =* 0.048; Figure 2C).

### 2.4. H.E. Normalizes Metabolic Pathways Related to Dopamine-Serotonin Neurotransmission

**Figure 3:**
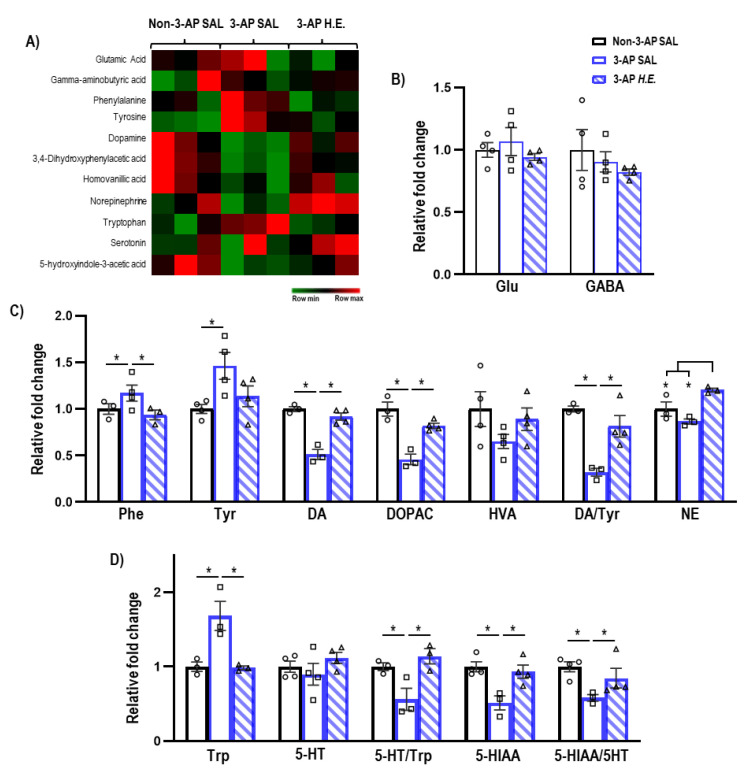
Mass spectrometry analysis of neurotransmitters and their metabolites in the cerebellum. (**A**) Heatmap of neurotransmitter expression in each group. (**B**) Relative expressions of glutamate and GABA. (**C**) Relative expressions of phenylalanine, tyrosine, dopamine, 3,4-dihydroxyphenylacetic acid (DOPAC), homovanillic acid (HVA), norepinephrine, and ratios of DOPAC/dopamine, HVA/dopamine, and dopamine/tyrosine. (**D**) Relative expressions of tryptophan, serotonin, 5-hydroxyindole-3-acetic acid (5-HITT), and ratios of serotonin/tryptophan and 5-HIAA/serotonin. Indicators: * *p*-values ≤ 0.05, ◯ Non-3-AP SAL group, ☐ 3-AP SAL group, △ 3-AP *H.E.* group.

Given that *H.E.* has been previously reported to modulate hippocampal neurotransmission [35], we next investigated the effect of *H.E.* treatment on neurotransmitter levels. The administration of 3-AP significantly increased phenylalanine (*p =* 0.047) and tyrosine (*p =* 0.005), and decreased dopamine (*p =* 0.014), DOPAC (*p =* 0.014), and the dopamine/tyrosine ratio (*p =* 0.014) compared to the control group (Figure 3C). Interestingly, *H.E.* treatment normalized phenylalanine, dopamine, DOPAC, and the dopamine/tyrosine ratio compared to the 3-AP group (all *p* < 0.047). Additionally, *H.E.* treatment also elevated norepinephrine levels compared to both 3-AP and control groups (all *p* < 0.014). No significant differences were found for HVA when comparing all groups (*p =* n.s.).

Administration of 3-AP increased tryptophan (*p =* 0.014) and decreased 5HIAA, 5-HT/Trp turnover, and 5HIAA/5-HT ratio compared to control animals (all *p* < 0.047) (Figure 3D). Remarkably, *H.E.* treatment normalized tryptophan, 5HIAA, 5-HT/Trp turnover, and 5HIAA/5-HT ratio (all *p* < 0.047). No significant differences in glutamate and GABA levels were detected when comparing all groups (*p* = n.s.; Figure 3B*).*

### 2.5. H.E. Normalizes Glycolysis-Related Metabolites

**Figure 4:**
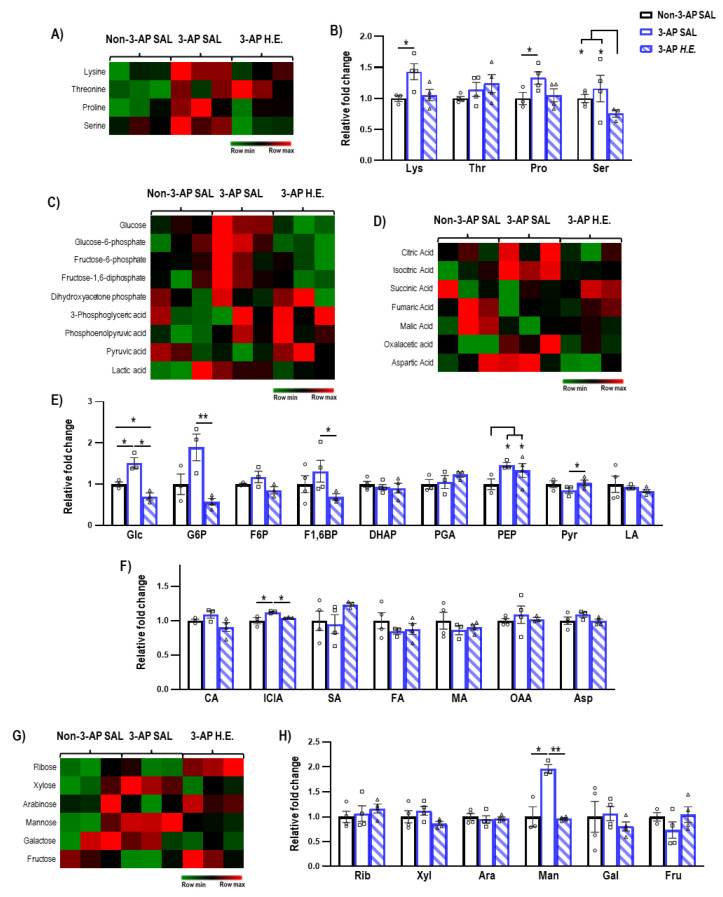
Mass spectrometry analysis of the metabolites changes in the cerebellum. (**A**,**B**) Heatmap and comparison of the expression of amino acids. (**C**,**E**) Heatmap and comparison of the expression of glycolysis-related metabolites, (**D**,**F**) TCA cycle-related metabolites, and (**G**,**H**) sugars. Indicators: * *p*-values ≤ 0.05, ** *p*-values ≤ 0.01, ◯ Non-3-AP SAL group, ☐ 3-AP SAL group, △ 3-AP *H.E.* group.

Next, we investigated the effects of *H.E.* treatment on metabolites related to amino acids (Figure 4B), glycolysis (Figure 4E), TCA cycle (Figure 4F), and sugars (Figure 4H). We found that *H.E.* significantly increased levels of lysine and proline in 3-AP treated animals compared to the control group (*p* < 0.047; Figure 4B). The *H.E.*+3-AP group also had reduced levels of serine compared to both control and 3-AP groups (all *p* < 0.047). No remarkable differences in threonine were detected when comparing all groups (*p =* n.s.). In 3-AP animals, we found altered respiratory pathway-related metabolites including increased levels of glucose (*p =* 0.014), phosphoenolpyruvic acid (*p =* 0.014), isocitric acid (*p =* 0.047), and mannose (*p =* 0.014) compared to the control group (Figure 4E–H). The *H.E.* treatment normalized the changes in glucose (*p =* 0.014), glucose-6-phosphate (*p =* 0.008), fructose-1,6-diphosphate (*p =* 0.047), pyruvic acid (*p =* 0.042), isocitric acid (*p =* 0.047), and mannose (*p =* 0.008) to levels similar to those in the controls. No remarkable changes were detected for ribose, xylose, arabinose, galactose, and fructose when comparing all groups (all *p =* n.s.; Figure 4H).

### 2.6. Correlational Analysis of Neuroinflammatory Genes in Cerebellar Tissues

**Figure 5:**
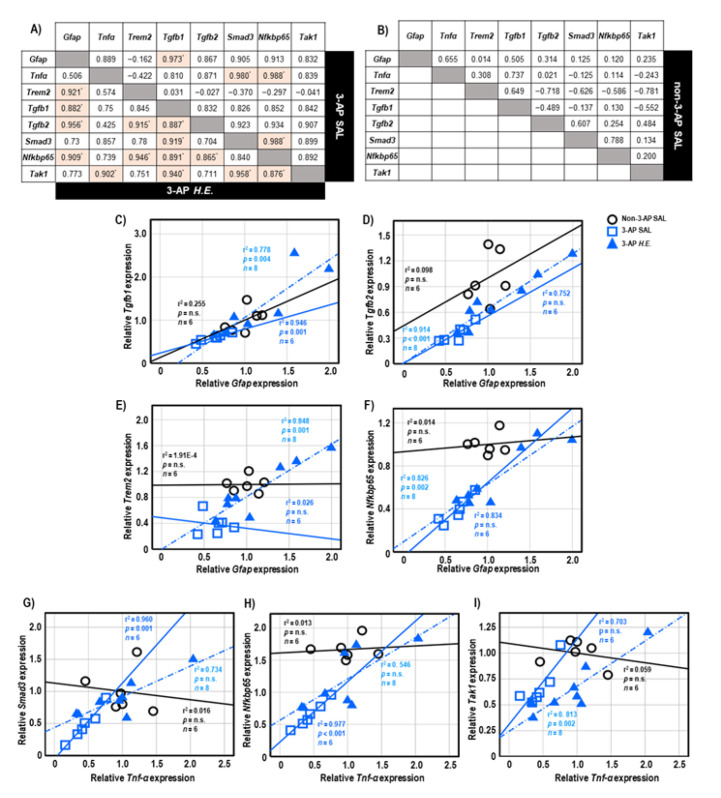
The tables show the gene correlations in the 3-AP group (upper-right), *H.E.*+3-AP group (Lower-left), and control group in (**A**,**B**). Scatter plots of significant correlations among groups by Spearman correlational analysis. Gene expression of *Gfap* was significantly correlated with the gene expression of (**C**) *Tgfb1*, (**D**) *Tgfb2*, (**E**)*Trem2*, and (**F**) *Nfkbp65*. Gene expression of *Tnf*-α was correlated with the gene expression of (**G**) *Smad3*, (**H**) *Nfkbp65*, and (**I**) *Tak1*. The *p* values were adjusted by Bonferroni correction for multiple comparisons. Indicators: * correlation was significant at *p* < 0.00625 for gene expression data. Indicators: ◯ Non-3-AP SAL group, ☐ 3-AP SAL group, △ 3-AP *H.E.* group.

**Figure 6:**
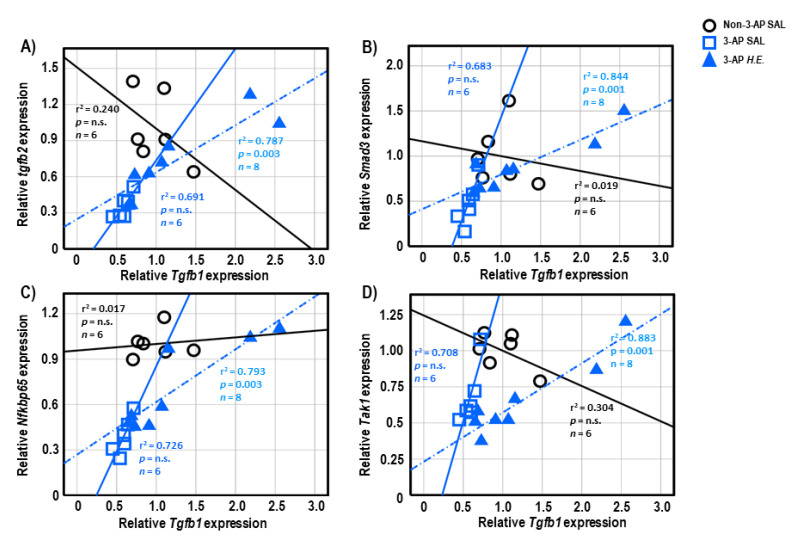

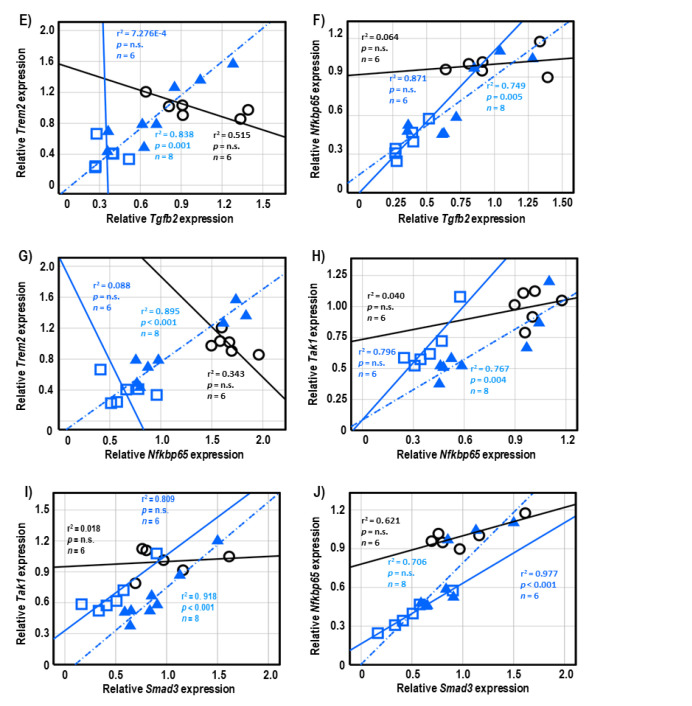
Spearman correlational analysis of genes showing significant differences after *H.E.* treatment. (**A**–**D**) Scatter plots represent significant correlations of the gene expression of *Tgfb1* with (**A**) *Tgfb2*, (**B**) *Smad3*, (**C**) *Nfkbp65*, and (**D**) *Tak1*. (**E**,**F**) Scatter plots represent correlations of the gene expression of *Tgfb2* with (**E**) *Trem2* and (**F**) *Nfkbp65*. (**G**,**H**) Scatter plots represent correlations of the gene expression of *Nfkbp65* with (**G**) *Trem2* and (**H**) *Tak1*. (**I**,**J**) Scatter plots represent correlations of the gene expression of *Smad3* with (**G**) *Tak1* and (**H**) *Nfkbp65*. Indicators: ◯ Non-3-AP SAL group, ☐ 3-AP SAL group, △ 3-AP *H.E.* group.

Both 3-AP and *H.E.*+3-AP groups showed a significant correlation between the expressions of *Gfap* and *Tgfb1* (all r^2^ < 0.946, *p* < 0.004; Figure 5A,C). We also observed significant correlations between *Gfap* and *Tgfb2* (r^2^ = 0.914, *p* < 0.001), *Trem2* (r^2^ = 0.848, *p =* 0.001), and *Nfkbp65* (r^2^ = 0.826, *p =* 0.002) in the *H.E.*+3AP group, but not in the 3-AP and control groups (Figure 5D–F). Spearman correlational analysis showed significant positive correlations between the expressions of *Tnf*-α, *Smad3* (r^2^ = 0.960, *p =* 0.001), and *Nfkbp65* (r^2^ = 0.977, *p* < 0.001) in the 3-AP group (Figure 5G,H). Compared to the 3-AP and control groups, the *H.E.*+3-AP group showed a significant correlation between *Tnf*-α and *Tak1* (r^2^ = 0.813, *p =* 0.002; Figure 5I); significant gene correlations of *Tgfb1* with *Tgfb2* (r^2^ = 0.787, *p =* 0.003), *Smad3* (r^2^ = 0.844, *p =* 0.001), *Nfkbp65* (r^2^ = 0.793, *p =* 0.003), and *Tak1* (r^2^ = 0.883, *p =* 0.001) (Figure 6A–D); significant gene correlations of *Tgfb2* with *Trem2* (r^2^ = 0.838, *p =* 0.001) and *Nfkbp65* (r^2^ = 0.749, *p =* 0.005) (Figure 6E,F); and significant gene correlations of *Nfkbp65* with *Trem2* (r^2^ = 0.895, *p* < 0.001) and *Tak1* (r^2^ = 0.767, *p =* 0.004) (Figure 6G,H). Additionally, we also found that the *H.E*+3-AP group had remarkable gene correlations between *Smad3* and *Tak1* (r^2^ = 0.918, *p* < 0.001), and the 3-AP group had gene correlations between *Smad3* and *Nfkbp65* (r^2^ = 0.977, *p* < 0.001) (Figure 6I,J). The control group showed no significant correlations among the analyzed genes, suggesting that 3-AP and *H.E.* treatments induced specific neuroinflammatory responses.

## 3. Discussion

*Hericium erinaceus* contains diverse compounds with neuroprotective activity [36]. Our previous study showed that the antidepressant effects of *H.E.* were attributed to several isolated compounds including adenosine, herierin III, and herierin IV [32]. The study showed that 25 mg/kg *H.E.* also has good neuroprotective potential [32]. The neurotoxin 3-AP is a nicotinamide antagonist targeting the inferior olivary nucleus and has been extensively used to generate animal models of cerebellar ataxia. A study has shown that 3-AP reduced the density of dendritic spines of Purkinje cells and decreased the expressions of AMPA and PSD-95 in the cerebellar cortex, suggesting that 3-AP-induced ataxic-like motor impairments associated with an alteration in the morphology and connectivity of Purkinje cells [37]. In the present study, we initially used a dose of 65 mg/kg 3-AP based on other previous studies [38,39], but none of the rats survived within a day after intraperitoneal injection. We performed a pilot study to optimize the dose of 3-AP, which showed that a dose of 40 mg/kg 3-AP could generate rats with ataxia accompanied by balance and motor impairments. Based on previous publications and our pilot studies, we selected a dose of 40 mg/kg 3-AP and 25 mg/kg *H.E.* in this study.

Our previous findings also demonstrated that 25 mg/kg *H.E.* could potentially rescue motor impairments through neuroprotective mechanisms involving ERK-CREB-PSD95 signaling in a 3-AP rat model of ataxia [19]. In the present study, we further investigated the molecular mechanisms of the effects of *H.E.* on neuroinflammation, neurotransmission, and energy-related metabolites in an animal model of ataxia. We showed that animals administered 3-AP had significant body weight loss compared to non-3-AP control animals. The *H.E.*+3-AP group progressively showed improved body weight compared to the 3-AP group. The normalization of body weight in *H.E.*+3-AP animals could be explained by the restoration of the lost appetite induced by 3-AP stress, or by the general improvement in motor coordination by the *H.E.* treatment. According to our previous data (Figure S7), rats in the 3-AP group remained ataxic with signs of motor impairment throughout the duration of the study of 21, following a single 3-AP injection without showing significant improvement compared to the control group. However, due to the timeframe of our experimental design, it is uncertain if the motor impairment induced by 3-AP is definitive. Moreover, animals, like humans, show spontaneous recovery; therefore, it can be difficult to pinpoint and evaluate effects of 3-AP. After the behavioral data from the accelerated rotarod and rod tests were normalized to the body weight of the respective animals, we observed that *H.E.* treatment significantly improved motor coordination and balance functions in 3-AP animals.

At 3 weeks after the 3-AP injection, we did not observe any significant changes in the expression of *Iba1, Il1b*, *Il18,* or *iNos*, indicating no microglial and pro-inflammatory responses during the chronic phase of ataxia. This phenomenon could be explained by a natural compensatory mechanism of the animal’s immune defense system. However, the 3-AP group showed downregulated expressions of pro-inflammatory *Gfap*, *Tnf*-α, and *Nfkbp65* genes, which were rescued by the *H.E.* treatment to levels comparable to those of the control group, suggesting normal levels of these genes participate in neuron protection and damage recovery [40,41]. Despite tremendous literature showing the aversive role of pro-inflammatory-related genes, emerging studies have shown that elevated pro-inflammatory cytokines are involved in both neurodegeneration and neuroprotection [42]. For example, GFAP, a classical marker of astrocytes, plays pivotal roles in neuronal regeneration, repair, structural support, nutritional supply, and synaptic transmission [43,44]. It has been reported that the downregulation of *Gfap* mRNA was associated with inhibited astroglial growth and function [45]. In addition, although TNF-α and NF-κB have been associated with pro-inflammatory responses [46], recent studies have suggested that TNF-α has anti-inflammatory [47], neuroprotective [48], and anti-apoptotic activities through the modulation of the NF-κB pathway [49]. Overall, our results showed that *H.E.* treatment can rescue the downregulated expression of *Gfap, Tnf*-α, and *Nfkb65* back to the levels in the controls, which is important for regulating inflammatory response and neuronal survival.

Treatment with 3-AP remarkably downregulated the expression of *Trem2*, *Tgfb1*, *Tgfb2*, *Smad2, Smad3, Tak1,* and *Jmjd3* in rat cerebellum. Treatment with *H.E.* normalized the expression of *Trem2*, *Tgfb1*, *Tgfb2,* and *Smad3* back to the levels in the control group, whereas the expression of *Smad2, Jmjd3, and Tak1* were not rescued. It has been shown that TREM2 participates in the anti-inflammatory response [50]. Furthermore, TGF-β was reported to participate in both anti- and pro-inflammatory responses [51], whereas impaired TGF-β signaling was associated with a dysregulated microglial response [52]. Hence, the regulation of TGF-β signaling by *H.E.* could potentially contribute to the restoration of normal immune function. Besides pro- and anti-inflammatory roles, the TGF-β/Smad3 pathway is also involved in other neuroprotective mechanisms including anti-apoptosis, neurogenesis, and neurotransmission [53,54,55]. Therefore, the improvement in motor coordination by *H.E.* could potentially be mediated through the canonical TGF-β/Smad3 signaling pathway. Furthermore, the dysregulated anti-inflammatory response in 3-AP-treated animals is potentially due to a negative relationship between the downregulated *Tgfb2* expression and poor motor balance in the rod test when compared to both the control and *H.E.*+3-AP groups. However, due to the small sample size, this relationship needs to be further verified.

A previous study demonstrated that patients with cerebellar ataxia had mitochondrial COX-IV deficiency [56]. After 3 weeks of *H.E.* treatment, we observed the significantly upregulated protein expression of COX-IV, suggesting that *H.E*. can rescue neuronal respiratory chain deficits and mitochondrial dysfunction [57]. We also observed a significant downregulation of the pro-apoptotic protein BAX [58] in both 3-AP and *H.E.*+3-AP animals, suggesting a general compensatory anti-apoptotic regulatory mechanism in response to the chronic neuronal loss induced by 3-AP. In addition, our data showed that *H.E.* significantly increased Akt activity and the pAkt/Akt ratio. Although a recent report suggests that Akt has multiple neuroprotective effects in DNA repair and cell survival by inhibiting neuronal death [59], our findings indicate that Akt signaling also has potential roles in mitochondrial and apoptotic regulation [34,60]. This result further supports our previous finding that an increase in Akt enhances the phosphorylation of CREB, which inhibits the expression of caspase 3, an apoptotic marker of the degradation of cytoskeletal proteins [19] and a regulator of mitochondrial function and neurotransmission [57].

The administration of 3-AP upregulated levels of phenylalanine and tyrosine, but downregulated dopamine, DOPAC, and the dopamine/tyrosine ratio in 3-AP animals. This suggests that the impairment of tyrosine hydroxylase could affect the conversion of phenylalanine/tyrosine to dopamine and the turnover of dopamine and its metabolites [57,61]. Additionally, 3-AP treatment increased tryptophan but decreased 5-HIAA, 5-HT/tryptophan turnover, and the 5-HIAA/5HT ratio, indicating the impairment of serotonin conversion and its metabolic pathways [57,61]. Treatment with *H.E.* rescued dopamine and serotonin conversion and metabolic pathways back to the levels in the controls. These results are in line with a study by Chiu et al., which found that an *H.E.* bioactive compound, erinacine A, restored the decreased levels of a monoamine neurotransmitter in stressed mice [62]. Taken together, our findings suggest that *H.E.* treatment can potentially rescue the dysregulated dopamine and serotonin metabolic pathways in the 3-AP-induced ataxia model.

We found 3-AP administration also induced a state of hypermetabolism, as demonstrated by significantly increased levels of glucose, glucose-6-phosphate, fructose-1,6-diphosphate, phosphoenolpyruvic acid, and isocitric acid. These findings are in line with a study that reported an association between glucose hypermetabolism and neurodegeneration in amyotrophic lateral sclerosis [63]. Treatment with *H.E.* restored the hypermetabolic state in 3-AP-treated animals to a level comparable to that of the control animals. We also observed elevated levels of amino acids and sugars in 3-AP animals, which were also normalized by *H.E.* treatment back to the levels in the controls.

To further investigate the effects of *H.E.* on the neuroinflammatory response, we performed a correlational analysis to study the gene-gene interactions and relationships [31,64]. We found significant positive correlations in the gene expressions of *Tnf*-α with *Smad3* and *Nfkbp65* in the 3-AP group, but not in the *H.E.*+3-AP or control groups. These results suggest that the reduced expression of *Tnf*-α was mediated by the reduced expression of *Smad3* and *Nfkbp65* in 3-AP treated animals, which could be restored by the *H.E.* treatment. Additionally, we also found positive correlations in the gene expression of *Gfap* with *Trem2, Tgfb1*, *Tgfb2*, and *Nfkbp65* in the *H.E.*+3-AP group but not in the 3-AP or control groups, which suggests that the anti-inflammatory effects of *H.E.* involve the interaction of these genes [44,55].

## 4. Materials and Methods

### 4.1. Source and Composition of H.E.

In this study, the standardized aqueous extract of *H.E.* (Nev-Gro^®^, Batch No. 7H2308X, Ganofarm R&D Private Limited, Tanjung Sepat, Selangor, Malaysia) was used for investigating the mechanisms related to the changes of pro- or anti-inflammatory markers and the regulation of metabolites in cerebellar ataxia model. Fresh fruiting bodies of *H.E.* were boiled in reverse osmosis water for 4 h, filtered, concentrated, and spray-dried. The standardized aqueous extract has a defined content of beta 1,3–1,6 glucan (20.66%) and adenosine (0.17%) (Nova Laboratories Private Limited, Sepang, Selangor, Malaysia). We isolated and identified three major compounds from *H.E.,* namely adenosine (Supplementary Figures S1 and S2, Table S1), herierin III (Supplementary Figures S3 and S4, Table S2) and Herierin IV (Supplementary Figures S5 and S6, Table S3) [32]. The detailed information regarding the isolation and identification of compounds from *H.E.* were previously reported by our laboratory (see Chong et al. 2021) [32].

### 4.2. Subjects

The animal protocols were approved by the Committee on the Use of Live Animals in Teaching and Research (CULATR No. 4495.17) of the University of Hong Kong. Ten-week-old male *Sprague Dawley* rats (*n* = 25) were housed in pairs under standard conditions (temperature: 25–27 °C, humidity: 60–65%, 12-h light/dark cycle) with food and water available *ad libitum*. Rats were subject to training before the motor behavioral tests. After ensuring that there were no significant differences in their motor performance, rats were randomly assigned into the non-3-AP control group (*n* = 8) and 3-AP treatment groups. Any non-responders to the 3-AP injection were removed from the experiment before the animals were further assigned into the 3-AP (*n* = 9) and *H.E.*+3-AP (*n* = 8) groups.

### 4.3. Administration of 3-AP and H.E.

Both 3-AP and *H.E.*+3-AP rats were administered with a single intraperitoneal injection of 3-AP (40 mg/kg body weight; Sigma-Aldrich, Missouri, USA). After 2 days, *H.E.* (25 mg/kg) or saline was administered intraperitoneally to the *H.E.*+3-AP group or 3-AP group once daily for 21 days. Whereas non-3-AP control rats received a single intraperitoneal injection of 0.9% NaCl saline solution. After 2 days, saline was administered intraperitoneally to the group once daily for 21 days.

### 4.4. Body Weight and Behavioral Tests

Behavioral data from the accelerated rotarod test and rod test were obtained from a previous experiment [19] and were reanalyzed accordingly. For the accelerated rotarod test, the percentage deficit of the total time on the rod (latency to fall) was calculated as:

$$\left(X-Baseline\right)/\left(X\;+\;Baseline\right)\;\times \;100\%.$$

where *X* refers to the latency to fall in the current test and baseline refers to the latency to fall during the rotarod test before 3-AP administration.

For the rod test, the total time on the rod (latency to fall) within 5 min was recorded. The animal’s body weight was measured before each behavioral test and the behavioral data were normalized to the body weight of the respective animals. All rats were trained and screened for functional mobility prior to the actual test. Rats that were uncooperative or refused to learn were excluded from the experiment.

### 4.5. Tissue and Histological Processing

At 21 days after *H.E.* or saline administration, rats were euthanized by sodium pentobarbital (Dorminal, Alfasan International BV, Woerden, Holland) and perfused with 0.9% NaCl saline solution. Rats were decapitated and their brains were extracted and dissected into two halves. One half of the brain was post-fixed in 4% paraformaldehyde fixative solution for 1 day and then immersed in 15% and 30% buffered sucrose solution, and subsequently snap-frozen in liquid nitrogen and stored at −80 °C. The other half of the brain was immediately snap-frozen in liquid nitrogen and stored at −80 °C. Cerebellar regions were macrodissected for gene and protein analysis. The experimental procedures were conducted as previously described [19,65].

### 4.6. Gene Expression Study

Quantitative real-time PCR (qPCR) was performed on cerebellar tissue as previously described [19,66,67]. TRIZOL (Life Technologies, Carlsbad, CA, USA) was added to the dissected brain tissue to isolate total RNA. The isolated RNA was converted to cDNA using a cDNA synthesis kit (Takara Bio Inc., Shiga, Japan). Next, qPCR was performed to analyze inflammation-related genes including glial fibrillary acidic protein *(Gfap*), allograft inflammatory factor 1 *(Iba1)*, interleukin 1 beta *(Il1b)*, tumor necrosis factor alpha *(Tnf*-α*)*, interleukin 18 *(Il18)*, nitric oxide synthase, inducible *(iNos)*, interleukin 6 *(Il-6)*, triggering receptor expressed on myeloid cell *(Trem2)*, transforming growth factor beta 1 (*Tgfb1*) and 2 *(Tgfb2)*, mothers against decapentaplegic homolog 2 *(Smad2)* and 3 *(Smad3)*, nuclear factor kappa B p65 *(Nfkbp65)*, TGFB Activated Kinase 1 *(Tak1)*, jumonji domain-containing protein D3 *(Jmjd3)*, glycogen synthase kinase 3-beta *(Gsk3b)*, and hypoxanthine phosphoribosyltransferase (*Hprt)*. The qPCR was performed in duplicate on well plates (Micro-Amp Optia 384 Reaction Plate, Thermo Fisher Scientific, Waltham, MA, USA) under standard conditions (50 °C for 2 min, 95 °C for 10 min, and 40 cycles of 95 °C for 10 s and 60 °C for 30 s) using the StepOnePlus Real-time PCR system (Thermo Fisher Scientific, Waltham, MA, USA). The PCR products were monitored by SYBR Green quantitative PCR mix (Applied Biosystems, Warrington, UK) and analyzed by the StepOne Real-Time PCR software (v2.3). Gene expression was normalized to the reference gene *Hprt* using the ratio 2^−ΔΔC^_T_ method and presented as the relative gene expression against the control group. The primer sequences of the analyzed genes are listed in Table 1.

### 4.7. Western Blot Analysis

Western blot analysis was performed on cerebellar tissues according to our previously published studies [19,68]. The dissected cerebellar tissues were homogenized in RIPA buffer with protease and phosphatase inhibitors (Thermo Scientific, Rockford, IL, USA). The protein concentration of each protein lysate sample was measured by a Bio-Rad DC Protein Assay Kit (Bio-Rad Laboratories, Inc., Hercules, CA, USA). Protein lysate diluted in lysis buffer was separated by 12% SDS-PAGE and transferred onto PVDF membranes by semi-dry electroblotting. Membranes were blocked with 5% milk in tris-buffered saline (TBS) for 1 h at room temperature, followed by incubation with the primary antibodies overnight at 4 °C. The primary antibodies included Akt, pAkt, COX-IV, VDAC, GAPDH, BAX, Lamin B1 (1:1000; Cell Signaling Technology, Inc., Beverly, MA, USA), and PDHe1α (1:1000; Abcam, Cambridge, MA, USA). After incubation overnight, membranes were rinsed with TBS in 0.1% Tween 20 (TBST) and then incubated with horseradish peroxidase-conjugated anti-mouse or anti-rabbit immunoglobin G secondary antibodies (1:2000; Invitrogen, Thermo Fisher Scientific, Waltham, MA, USA) for 2 h at room temperature. The protein bands were visualized using a Clarity Western ECL Substrate kit (Bio-Rad Laboratories, Inc., Hercules, CA, USA). The protein expression was normalized against the GAPDH loading control and presented as the relative protein expression against the control group.

### 4.8. Mass Spectrometry

Mass spectrometry analysis of the dissected cerebellar tissues was performed as previously described [66,67]. Two samples from the same group were randomly pooled, with each group consisting of three to four pools per group. The tissue sample (50 mg) was mixed with 500 μL methanol/water (80%, *v*/*v*) containing 200 ng norvaline as the internal control. The tissue sample was homogenized by two cycles of sonication at 10 microns for 20 s on ice with a 10-s interval. The sample was vortexed in 250 μL of 0.1 M HCl for 30 s and 400 μL chloroform was added. The sample was agitated for 15 min and centrifuged at 16,000× *g* for 5 min at 4 °C. The supernatant (375 μL) was dried under a gentle stream of nitrogen at room temperature. The dried residue was subject to derivatization in 40 μL methoxylamine hydrochloride (30 mg/mL in pyridine) for 2 h at room temperature. The trimethylsilyation step was performed using 70 μL MSFTA and 1% TMCS. The sample (1 µL) was analyzed by GC-MS/MS on an Agilent 7890B GC—Agilent 7010 Triple Quadrupole Mass Spectrometer system (Santa Clara, CA, USA). The sample was separated on an Agilent DB-5MS capillary column (30 m × 0.25 mm ID, 0.25 µm film thickness) under a constant flow rate of 1 mL/min. The GC oven program was started at 60 °C (hold time 1 min) and then increased at a rate of 10 °C/min to 120 °C, 3 °C/min to 150 °C, 10 °C/min to 200 °C, and finally 30 °C/min to 280 °C (hold time 5 min). The inlet temperature and transfer line temperature were 250 °C and 280 °C, respectively. The characteristic quantifier and qualifier transitions were monitored in the MRM mode. The mass spectra were acquired in SCAN mode from *m*/*z* 50–500. Data analysis was performed using the Agilent MassHunter Workstation Quantitative Analysis Software 12.0. Linear calibration curves for each analyte were generated by plotting the peak area ratio of the external/internal standard against the standard at different concentrations. Analytes were confirmed by comparing the retention time and ratio of characteristic transitions between the sample and standard during the run.

### 4.9. Statistical Analysis

Statistical analysis was performed using IBM SPSS Statistics 27. A Shapiro–Wilk test was used to examine the normality of data distribution. Normally distributed data were analyzed by one-way ANOVA with an LSD *post hoc* test. For non-normally distributed data, a non-parametric Kruskal–Wallis or Mann–Whitney test was used for multiple comparisons, as appropriate. A non-parametric test was used to analyze the mass spectrometry results of the pooled samples. All data were presented as mean ± S.E.M. A *p*-value ≤ 0.05 was considered statistically significant. Spearman correlation coefficients with Bonferroni correction were applied to investigate the relationship between weight-normalized behavior and genes showing significant differences between groups. Variables showing significance after the Bonferroni correction were presented by scatter plot with each individual data point plotted with 95% confidence intervals.

## 5. Conclusions

In conclusion, our results demonstrate that *H.E.* treatment can rescue motor coordination and balance deficits in rats with 3-AP-induced cerebellar ataxia. The effect of *H.E.* on restoring the motor impairment potentially involves TGF-β/Smad3 signaling via enhancing mitochondrial and Akt activity, normalizing monoaminergic conversion, and restoring metabolic pathways and hypermetabolic status. Our findings support the use of *H.E.* as a novel approach for treating ataxia symptoms. Importantly, ethical concerns on the use of *H.E.* in patients with ataxia need to be considered in the future clinical applications. Moreover, collaborations between various international funding bodies and interdisciplinary research are needed to enhance the development of rapid and robust therapies [69,70].

## Figures and Tables

**Table 1:** The primer sequences used in the real-time quantitative PCR.

Genes	Primer Sequences
***Gfap***	Forward (5′-3′): CCTTGAGTCCTTGCGCGGC
	Reverse (5′-3′): TTGGCCCTCCTCCTCCAGC
***Iba1***	Forward (5′-3′): GAAGCGAATGCTGGAGAAAC
	Reverse (5′-3′): CCTCCAATTAGGGCAACTCA
***Il1b***	Forward (5′-3′): CACCTCTCAAGCAGAGCACAG
	Reverse (5′-3′): GGGTTCCATGGTGAAGTCAAC
***Tnf-a***	Forward (5′-3′): AAATGGGCTCCCTCTCATCAGTTC
	Reverse (5′-3′): TCTGCTTGGTGGTTTGCTACGAC
***Il18***	Forward (5′-3′): ATATCGACCGAACAGCCAAC
	Reverse (5′-3′): TGGCACACGTTTCTGAAAGA
***iNos***	Forward (5′-3′): GCACAGAGGGCTCAAAGG
	Reverse (5′-3′): CACATCGCCACAAACATAAA
***Il6***	Forward (5′-3′): TCCTACCCCAACTTCCAATGCTC
	Reverse (5′-3′): TTGGATGGTCTTGGTCCTTAGCC
***Trem2***	Forward (5′-3′): AACTTCAGATCCTCACTGGACCC
	Reverse (5′-3′): GCAGAACAGAAGTCTTGGTGG
***Tgfb1***	Forward (5′-3′): TGGCGTTACCTTGGTAACC
	Reverse (5′-3′): GGTGTTGAGCCCTTTCCAG
***Tgfb2***	Forward (5′-3′): TCGACATGGATCAGTTTATGCG
	Reverse (5′-3′): CCCTGGTACTGTTGTAGATGGA
***Smad2***	Forward (5′-3′): ATGTCGTCCATCTTGCCATTC
	Reverse (5′-3′): AACCGTCCTGTTTTCTTTAGCTT
***Smad3***	Forward (5′-3′): AAGAAGCTCAAGAAGACGGGG
	Reverse (5′-3′): CAGTGACCTGGGGATGGTAAT
***Nfkb1***	Forward (5′-3′): GCGAGAGAAGCACAGATACCA
	Reverse (5′-3′): GGTCAGCCTCATAGTAGCCAT
***Tak1***	Forward (5′-3′): AGAGGTTGTCGGAAGAGGAGCTT
	Reverse (5′-3′): ACAACTGCCGGAGCTCCACAA
***Jmjd3***	Forward (5′-3′): CAACTCCATCTGGCTGTTACTG
	Reverse (5′-3′): CCTTCTGCAACCAATTCCAG
***Gsk3b***	Forward (5′-3′): CGGGACCCAAATGTCAAACT
	Reverse (5′-3′): CGTGACCAGTGTTGCTGAGT

## Data Availability

The original contributions presented in the study are included in the article, further inquiries can be directed to the corresponding authors.

## Supplementary Materials

The following supporting information can be downloaded at: https://www.mdpi.com/article/10.3390/ijms24076089/s1.

## References

[1] Klockgether T., Paulson H. (2011). Milestones in ataxia. Mov. Disord..

[2] Marsden J., Harris C. (2011). Cerebellar ataxia: Pathophysiology and rehabilitation. Clin. Rehabil..

[3] Jaques C.S., Escorcio-Bezerra M.L., Pedroso J.L., Barsottini O.G.P. (2021). The Intersection Between Cerebellar Ataxia and Neuropathy: A Proposed Classification and a Diagnostic Approach. Cerebellum.

[4] Kansal K., Yang Z., Fishman A.M., Sair H.I., Ying S.H., Jedynak B.M., Prince J.L., Onyike C.U. (2016). Structural cerebellar correlates of cognitive and motor dysfunctions in cerebellar degeneration. Brain.

[5] Ormerod I.E., Harding A.E., Miller D.H., Johnson G., MacManus D., Du Boulay E.P., Kendall B.E., Moseley I.F., McDonald W.I. (1994). Magnetic resonance imaging in degenerative ataxic disorders. J. Neurol. Neurosurg. Psychiatry.

[6] Shakkottai V.G., Costa M.D.C., Dell’Orco J.M., Sankaranarayanan A., Wulff H., Paulson H.L. (2011). Early Changes in Cerebellar Physiology Accompany Motor Dysfunction in the Polyglutamine Disease Spinocerebellar Ataxia Type 3. J. Neurosci..

[7] Rinaldo L., Hansel C. (2010). Ataxias and cerebellar dysfunction: Involvement of synaptic plasticity deficits?. Funct. Neurol..

[8] Yang Y., Sun K., Liu W., Zhang L., Peng K., Zhang S., Li S., Yang M., Jiang Z., Lu F. (2018). Disruption of Tmem30a results in cerebellar ataxia and degeneration of Purkinje cells. Cell Death Dis..

[9] Hoxha E., Balbo I., Miniaci M.C., Tempia F. (2018). Purkinje Cell Signaling Deficits in Animal Models of Ataxia. Front. Synaptic Neurosci..

[10] Ghorbani Z., Abdollahifar M.A., Vakili K., Moghaddam M.H., Mehdizadeh M., Marzban H., Rasoolijazi H., Aliaghaei A. (2022). Melittin administration ameliorates motor function, prevents apoptotic cell death and protects Purkinje neurons in the rat model of cerebellar ataxia induced by 3-Acetylpyridine. Toxicon.

[11] Horn K.M., Deep A., Gibson A.R. (2012). Progressive limb ataxia following inferior olive lesions. J. Physiol..

[12] Sarva H., Shanker V.L. (2014). Treatment Options in Degenerative Cerebellar Ataxia: A Systematic Review. Mov. Disord. Clin. Pract..

[13] Rai S.N., Mishra D., Singh P., Vamanu E., Singh M. (2021). Therapeutic applications of mushrooms and their biomolecules along with a glimpse of in silico approach in neurodegenerative diseases. Biomed. Pharmacother..

[14] Scuto M., Di Mauro P., Ontario M.L., Amato C., Modafferi S., Ciavardelli D., Salinaro A.T., Maiolino L., Calabrese V. (2019). Nutritional Mushroom Treatment in Meniere’s Disease with Coriolus versicolor: A Rationale for Therapeutic Intervention in Neuroinflammation and Antineurodegeneration. Int. J. Mol. Sci..

[15] Phang M.W.L., Lew S.Y., Chung I., Lim W.K.-S., Lim L.W., Wong K.H. (2021). Therapeutic roles of natural remedies in combating hereditary ataxia: A systematic review. Chin. Med..

[16] Lew S.Y., Phang M.W.L., Chong P.S., Roy J., Poon C.H., Yu W.S., Lim L.W., Wong K.H. (2022). Discovery of Therapeutics Targeting Oxidative Stress in Autosomal Recessive Cerebellar Ataxia: A Systematic Review. Pharmaceuticals.

[17] Yanshree, Yu W.S., Fung M.L., Lee C.W., Lim L.W., Wong K.H. (2022). The Monkey Head Mushroom and Memory Enhancement in Alzheimer’s Disease. Cells.

[18] Yang P., Lin C., Lin T., Chiang W. (2020). Hericium erinaceus Mycelium Exerts Neuroprotective Effect in Parkinson’s Disease-in vitro and in vivo Models. J. Drug Res. Dev..

[19] Chong P.S., Khairuddin S., Tse A.C.K., Hiew L.F., Lau C.L., Tipoe G.L., Fung M.-L., Wong K.H., Lim L.W. (2020). *Hericium erinaceus* potentially rescues behavioural motor deficits through ERK-CREB-PSD95 neuroprotective mechanisms in rat model of 3-acetylpyridine-induced cerebellar ataxia. Sci. Rep..

[20] Wang Z.Y., Fu H.T. (1981). Treatment of hereditary cerebellar ataxia with Ganoderma capense. Report of 4 cases. J. Tradit. Chin. Med..

[21] Hwang J.-H., Chen C.-C., Lee L.-Y., Chiang H.-T., Wang M.-F., Chan Y.-C. (2020). *Hericium erinaceus* enhances neurotrophic factors and prevents cochlear cell apoptosis in senescence accelerated mice. J. Funct. Foods.

[22] Lai P.-L., Naidu M., Sabaratnam V., Wong K.-H., David R.P., Kuppusamy U.R., Abdullah N., Malek S.N.A. (2013). Neurotrophic Properties of the Lion’s Mane Medicinal Mushroom, *Hericium erinaceus* (Higher Basidiomycetes) from Malaysia. Int. J. Med. Mushrooms.

[23] Roda E., Ratto D., De Luca F., Desiderio A., Ramieri M., Goppa L., Savino E., Bottone M.G., Locatelli C.A., Rossi P. (2022). Searching for a Longevity Food, We Bump into *Hericium erinaceus* Primordium Rich in Ergothioneine: The “Longevity Vitamin” Improves Locomotor Performances during Aging. Nutrients.

[24] Lew S.-Y., Yow Y.-Y., Lim L.-W., Wong K.-H. (2019). Antioxidant-mediated protective role of *Hericium erinaceus* (Bull.: Fr.) Pers. against oxidative damage in fibroblasts from Friedreich’s ataxia patient. Food Sci. Technol..

[25] Lew S.Y., Lim S.H., Lim L.W., Wong K.H. (2020). Neuroprotective effects of *Hericium erinaceus* (Bull.: Fr.) Pers. against high-dose corticosterone-induced oxidative stress in PC-12 cells. BMC Complement. Med. Ther..

[26] Li I.-C., Chang H.-H., Lin C.-H., Chen W.-P., Lu T.-H., Lee L.-Y., Chen Y.-W., Chen Y.-P., Chen C.-C., Lin D.P.-C. (2020). Prevention of Early Alzheimer’s Disease by Erinacine A-Enriched *Hericium erinaceus* Mycelia Pilot Double-Blind Placebo-Controlled Study. Front. Aging Neurosci..

[27] Tsai-Teng T., Chin-Chu C., Li-Ya L., Wan-Ping C., Chung-Kuang L., Chien-Chang S., Chi-Ying H.F., Chien-Chih C., Shiao Y.-J. (2016). Erinacine A-enriched *Hericium erinaceus* mycelium ameliorates Alzheimer’s disease-related pathologies in APPswe/PS1dE9 transgenic mice. J. Biomed. Sci..

[28] Lee K.-F., Tung S.-Y., Teng C.-C., Shen C.-H., Hsieh M.C., Huang C.-Y., Lee K.-C., Lee L.-Y., Chen W.-P., Chen C.-C. (2020). Post-treatment with erinacine A, a derived diterpenoid of H. erinaceus, attenuates neurotoxicity in MPTP model of Parkinson’s disease. Antioxidants.

[29] Kuo H.-C., Lu C.-C., Shen C.-H., Tung S.-Y., Hsieh M.C., Lee K.-C., Lee L.-Y., Chen C.-C., Teng C.-C., Huang W.-S. (2016). RETRACTED ARTICLE: *Hericium erinaceus* mycelium and its isolated erinacine A protection from MPTP-induced neurotoxicity through the ER stress, triggering an apoptosis cascade. J. Transl. Med..

[30] Ratto D., Corana F., Mannucci B., Priori E.C., Cobelli F., Roda E., Ferrari B., Occhinegro A., Di Iorio C., De Luca F. (2019). *Hericium erinaceus* Improves Recognition Memory and Induces Hippocampal and Cerebellar Neurogenesis in Frail Mice during Aging. Nutrients.

[31] Chong P.S., Fung M.L., Wong K.H., Lim L.W. (2020). Therapeutic Potential of *Hericium erinaceus* for Depressive Disorder. Int. J. Mol. Sci..

[32] Chong P.S., Poon C.H., Roy J., Tsui K.C., Lew S.Y., Phang M.W.L., Tan R.J.Y., Cheng P.G., Fung M.-L., Wong K.H. (2021). Neurogenesis-dependent antidepressant-like activity of *Hericium erinaceus* in an animal model of depression. Chin. Med..

[33] Vigna L., Morelli F., Agnelli G.M., Napolitano F., Ratto D., Occhinegro A., Di Iorio C., Savino E., Girometta C., Brandalise F. (2019). *Hericium erinaceus* Improves Mood and Sleep Disorders in Patients Affected by Overweight or Obesity: Could Circulating Pro-BDNF and BDNF Be Potential Biomarkers?. Evidence-Based Complement. Altern. Med..

[34] Bijur G.N., Jope R.S. (2003). Rapid accumulation of Akt in mitochondria following phosphatidylinositol 3-kinase activation. J. Neurochem..

[35] Brandalise F., Cesaroni V., Gregori A., Repetti M., Romano C., Orrù G., Botta L., Girometta C., Guglielminetti M.L., Savino E. (2017). Dietary Supplementation of *Hericium erinaceus* Increases Mossy Fiber-CA3 Hippocampal Neurotransmission and Recognition Memory in Wild-Type Mice. Evid.-Based Complement. Altern. Med..

[36] Li I.-C., Lee L.-Y., Tzeng T.-T., Chen W.-P., Chen Y.-P., Shiao Y.-J., Chen C.-C. (2018). Neurohealth Properties of *Hericium erinaceus* Mycelia Enriched with Erinacines. Behav. Neurol..

[37] González-Tapia D., Vázquez-Hernández N., Urmeneta-Ortiz F., Navidad-Hernandez N., Lazo-Yepez M., Tejeda-Martínez A., Flores-Soto M., González-Burgos I. (2021). 3-Acetylpyridine-induced ataxic-like motor impairments are associated with plastic changes in the Purkinje cells of the rat cerebellum. Neurología.

[38] Kaffashian M., Shabani M., Goudarzi I., Behzadi G., Zali A., Janahmadi M. (2011). Profound Alterations in the Intrinsic Excitability of Cerebellar Purkinje Neurons Following Neurotoxin 3-Acetylpyridine (3-AP)-Induced Ataxia in Rat: New Insights Into the Role of Small Conductance K+ Channels. Physiol. Res..

[39] Jiang Y.-Y., Cao B.-B., Wang X.-Q., Peng Y.-P., Qiu Y.-H. (2015). Cerebellar ataxia induced by 3-AP affects immunological function. Neuro Endocrinol. Lett..

[40] Sullivan P.G., Bruce-Keller A.J., Rabchevsky A.G., Christakos S., Clair D.K.S., Mattson M.P., Scheff S.W. (1999). Exacerbation of Damage and Altered NF-κB Activation in Mice Lacking Tumor Necrosis Factor Receptors after Traumatic Brain Injury. J. Neurosci..

[41] Scherbel U., Raghupathi R., Nakamura M., Saatman K.E., Trojanowski J.Q., Neugebauer E., Marino M.W., McIntosh T.K. (1999). Differential acute and chronic responses of tumor necrosis factor-deficient mice to experimental brain injury. Proc. Natl. Acad. Sci. USA.

[42] Wang W.-Y., Tan M.-S., Yu J.-T., Tan L. (2015). Role of pro-inflammatory cytokines released from microglia in Alzheimer’s disease. Ann. Transl. Med..

[43] Chiareli R.A., Carvalho G.A., Marques B.L., Mota L.S., Oliveira-Lima O.C., Gomes R.M., Birbrair A., Gomez R.S., Simão F., Klempin F. (2021). The Role of Astrocytes in the Neurorepair Process. Front. Cell Dev. Biol..

[44] Uddin S., Lim L.W. (2022). Glial cells in Alzheimer’s disease: From neuropathological changes to therapeutic implications. Ageing Res. Rev..

[45] Letournel-Boulland M.L., Fages C., Rolland B., Tardy M. (1994). Lipopolysaccharides (LPS), up-regulate the IL-1-mRNA and down-regulate the glial fibrillary acidic protein (GFAP) and glutamine synthetase (GS)-mRNAs in astroglial primary cultures. Eur. Cytokine Netw..

[46] Lawrence T. (2009). The Nuclear Factor NF-kappa B Pathway in Inflammation. Cold Spring Harb. Perspect. Biol..

[47] Zakharova M., Ziegler H.K. (2005). Paradoxical Anti-Inflammatory Actions of TNF-α: Inhibition of IL-12 and IL-23 via TNF Receptor 1 in Macrophages and Dendritic Cells. J. Immunol..

[48] Barger S.W., Hörster D., Furukawa K., Goodman Y., Krieglstein J., Mattson M.P. (1995). Tumor necrosis factors alpha and beta protect neurons against amyloid beta-peptide toxicity: Evidence for involvement of a kappa B-binding factor and attenuation of peroxide and Ca^2+^ accumulation. Proc. Natl. Acad. Sci. USA.

[49] Kim M., Jung K., Kim I.-S., Lee I.-S., Ko Y., Shin J.E., Park K.I. (2018). TNF-α induces human neural progenitor cell survival after oxygen–glucose deprivation by activating the NF-κB pathway. Exp. Mol. Med..

[50] Chen S., Peng J., Sherchan P., Ma Y., Xiang S., Yan F., Zhao H., Jiang Y., Wang N., Zhang J.H. (2020). TREM2 activation attenuates neuroinflammation and neuronal apoptosis via PI3K/Akt pathway after intracerebral hemorrhage in mice. J. Neuroinflamm..

[51] Sanjabi S., A Zenewicz L., Kamanaka M., A Flavell R. (2009). Anti-inflammatory and pro-inflammatory roles of TGF-β, IL-10, and IL-22 in immunity and autoimmunity. Curr. Opin. Pharmacol..

[52] Zöller T., Schneider A., Kleimeyer C., Masuda T., Potru P.S., Pfeifer D., Blank T., Prinz M., Spittau B. (2018). Silencing of TGFβ signalling in microglia results in impaired homeostasis. Nat. Commun..

[53] Dobolyi A., Vincze C., Pál G., Lovas G. (2012). The Neuroprotective Functions of Transforming Growth Factor Beta Proteins. Int. J. Mol. Sci..

[54] Song L., Liu F.-F., Liu C.-Y., Li X.-P., Zheng S.-Z., Li Q.-Q., Liu Q. (2015). Neuroprotective effects of SMADs in a rat model of cerebral ischemia/reperfusion. Neural Regen. Res..

[55] Hiew L.-F., Poon C.-H., You H.-Z., Lim L.-W. (2021). TGF-β/Smad Signalling in Neurogenesis: Implications for Neuropsychiatric Diseases. Cells.

[56] Lax N.Z., Hepplewhite P.D., Reeve A., Nesbitt V., McFarland R., Jaros E., Taylor R.W., Turnbull D.M. (2012). Cerebellar Ataxia in Patients With Mitochondrial DNA Disease: A molecular clinicopathological study. J. Neuropathol. Exp. Neurol..

[57] Wong K.Y., Roy J., Fung M.L., Heng B.C., Zhang C., Lim L.W. (2020). Relationships between Mitochondrial Dysfunction and Neurotransmission Failure in Alzheimer’s Disease. Aging Dis..

[58] Pawlowski J., Kraft A.S. (2000). Bax-induced apoptotic cell death. Proc. Natl. Acad. Sci. USA.

[59] Ahn J.-Y. (2014). Neuroprotection Signaling of Nuclear Akt in Neuronal Cells. Exp. Neurobiol..

[60] Yamaguchi H., Wang H.-G. (2001). The protein kinase PKB/Akt regulates cell survival and apoptosis by inhibiting Bax conformational change. Oncogene.

[61] Sesia T., Bulthuis V., Tan S., Lim L.W., Vlamings R., Blokland A., Steinbusch H.W., Sharp T., Visser-Vandewalle V., Temel Y. (2010). Deep brain stimulation of the nucleus accumbens shell increases impulsive behavior and tissue levels of dopamine and serotonin. Exp. Neurol..

[62] Chiu C.-H., Chyau C.-C., Chen C.-C., Lee L.-Y., Chen W.-P., Liu J.-L., Lin W.-H., Mong M.-C. (2018). Erinacine A-Enriched *Hericium erinaceus* Mycelium Produces Antidepressant-Like Effects through Modulating BDNF/PI3K/Akt/GSK-3β Signaling in Mice. Int. J. Mol. Sci..

[63] Zanovello M., Sorarù G., Campi C., Anglani M., Spimpolo A., Berti S., Bussè C., Mozzetta S., Cagnin A., Cecchin D. (2022). Brain Stem Glucose Hypermetabolism in Amyotrophic Lateral Sclerosis/Frontotemporal Dementia and Shortened Survival: An 18F-FDG PET/MRI Study. J. Nucl. Med..

[64] Hestermann D., Temel Y., Blokland A., Lim L.W. (2014). Acute serotonergic treatment changes the relation between anxiety and HPA-axis functioning and periaqueductal gray activation. Behav. Brain Res..

[65] Liu A., Jain N., Vyas A., Lim L.W. (2015). Ventromedial prefrontal cortex stimulation enhances memory and hippocampal neurogenesis in the middle-aged rats. eLife.

[66] Poon C.H., Liu Y., Pak S., Zhao R.C., Aquili L., Tipoe G.L., Leung G.K.-K., Chan Y.-S., Yang S., Fung M.-L. (2023). Prelimbic Cortical Stimulation with L-methionine Enhances Cognition through Hippocampal DNA Methylation and Neuroplasticity Mechanisms. Aging Dis..

[67] Tan S.Z.K., Neoh J., Lawrence A.J., Wu E.X., Lim L.W. (2020). Prelimbic Cortical Stimulation Improves Spatial Memory through Distinct Patterns of Hippocampal Gene Expression in Aged Rats. Neurotherapeutics.

[68] Yu W.S., Tse A.C.-K., Guan L., Chiu J.L.Y., Tan S.Z.K., Khairuddin S., Agadagba S.K., Lo A.C.Y., Fung M.-L., Chan Y.-S. (2022). Antidepressant-like effects of transcorneal electrical stimulation in rat models. Brain Stimul..

[69] Tan S.Z.K., Lim L.W. (2020). A practical approach to the ethical use of memory modulating technologies. BMC Med. Ethic.

[70] Tan S.Z.K., Zhao R.C., Chakrabarti S., Stambler I., Jin K., Lim L.W. (2021). Interdisciplinary Research in Alzheimer’s Disease and the Roles International Societies Can Play. Aging Dis..

