# Supplementary material for: Hericium erinaceus Promotes Anti-Inflammatory Effects and Regulation of Metabolites in an Animal Model of Cerebellar Ataxia
Source: Int J Mol Sci. 2023 Mar 23;24(7):6089. doi: 10.3390/ijms24076089 (PMC10094689; doi:10.3390/ijms24076089)

Supplementary Materials

Figure S1. ^{1}H NMR (MeOH-d_4, 600 MHz) spectrum of adenosine.

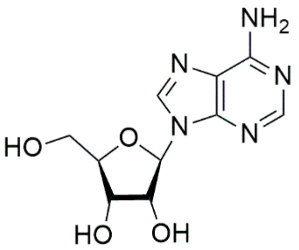

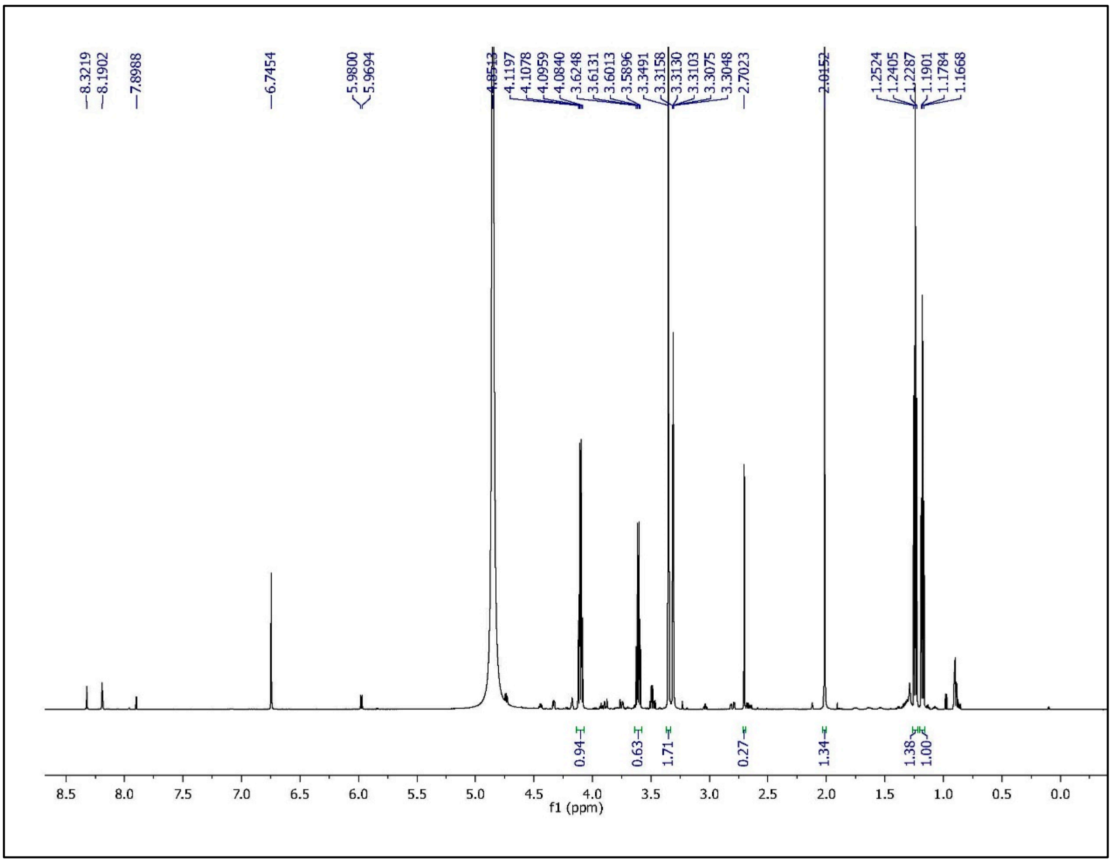

Figure S2. ^{13}C NMR (MeOH-d_4, 150 MHz) spectrum of adenosine.

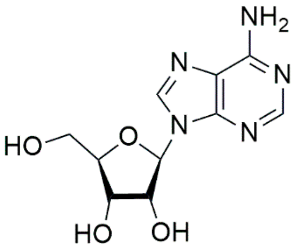

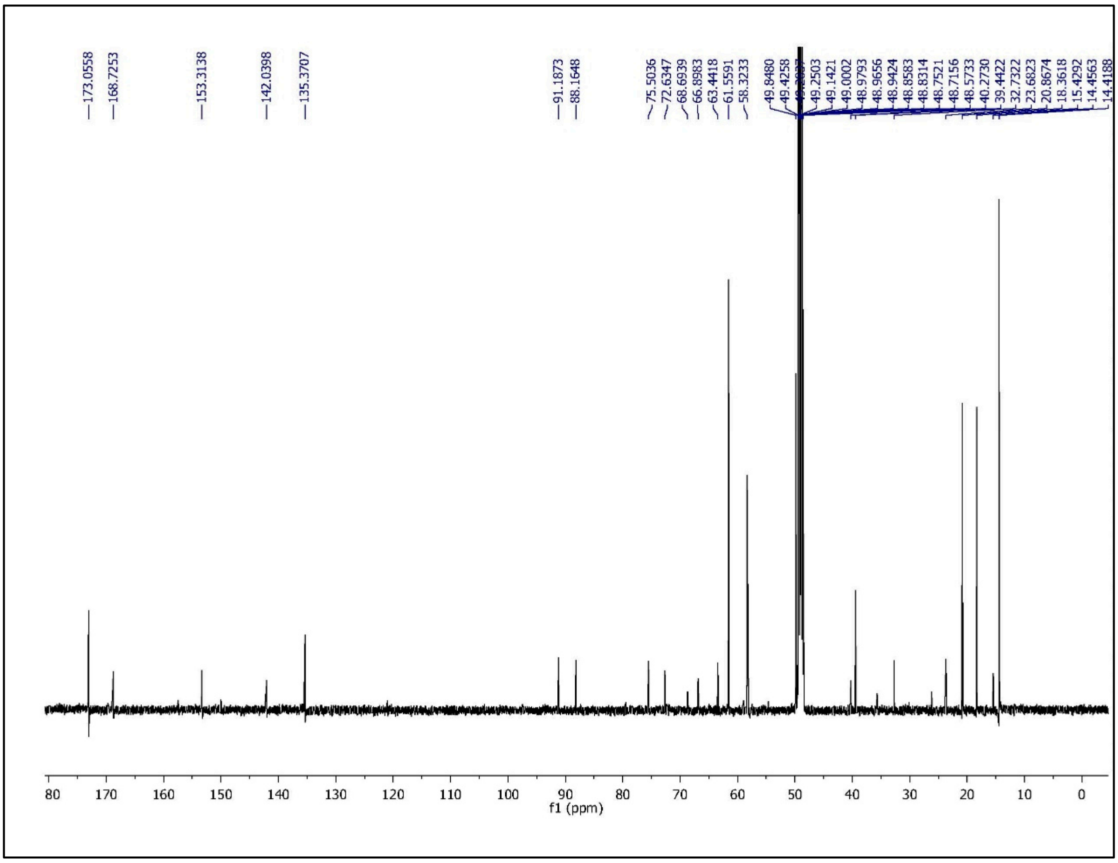

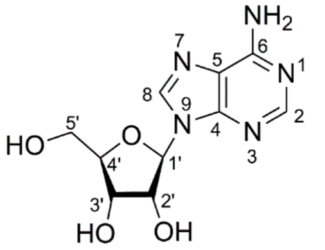

Table S1. ^{1}H and ^{13}C NMR spectroscopic data of adenosine (**1**).

Position	^{1}H (ppm)[1]	^{13}C (ppm)[2]
2	8.32, s	153.3
4		150.0
5		120.0
6		168.7
8	8.19, s	142.0
1’	5.97, d (6.4)	91.2
2’	4.43, br s	75.5
3’	4.40, br s	72.6
4’	4.21, br s	88.2
5’	3.80, m	63.4
NH_2	6.62, br s	

Assignments based on COSY, HSQC and HMBC. Recorded at 600 MHz in MeOH-d_4.[1]

Recorded at 150 MHz in MeOH-d_4.[2]

Figure S3. ^{1}H NMR (MeOH-d_4, 600 MHz) spectrum of herierin III.

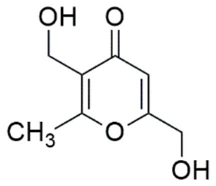

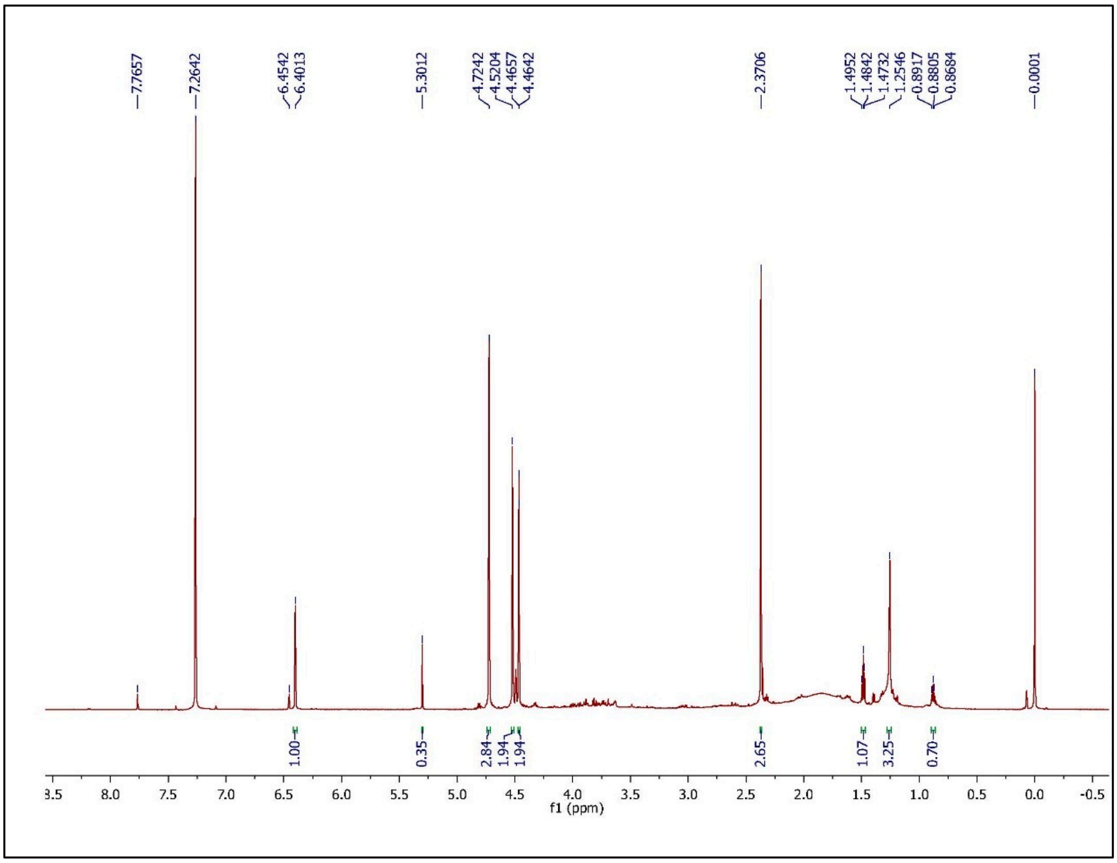

Figure S4. ^{13}C NMR (MeOH-d_4, 150 MHz) spectrum of herierin III.

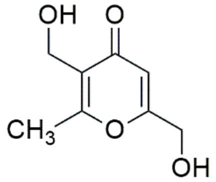

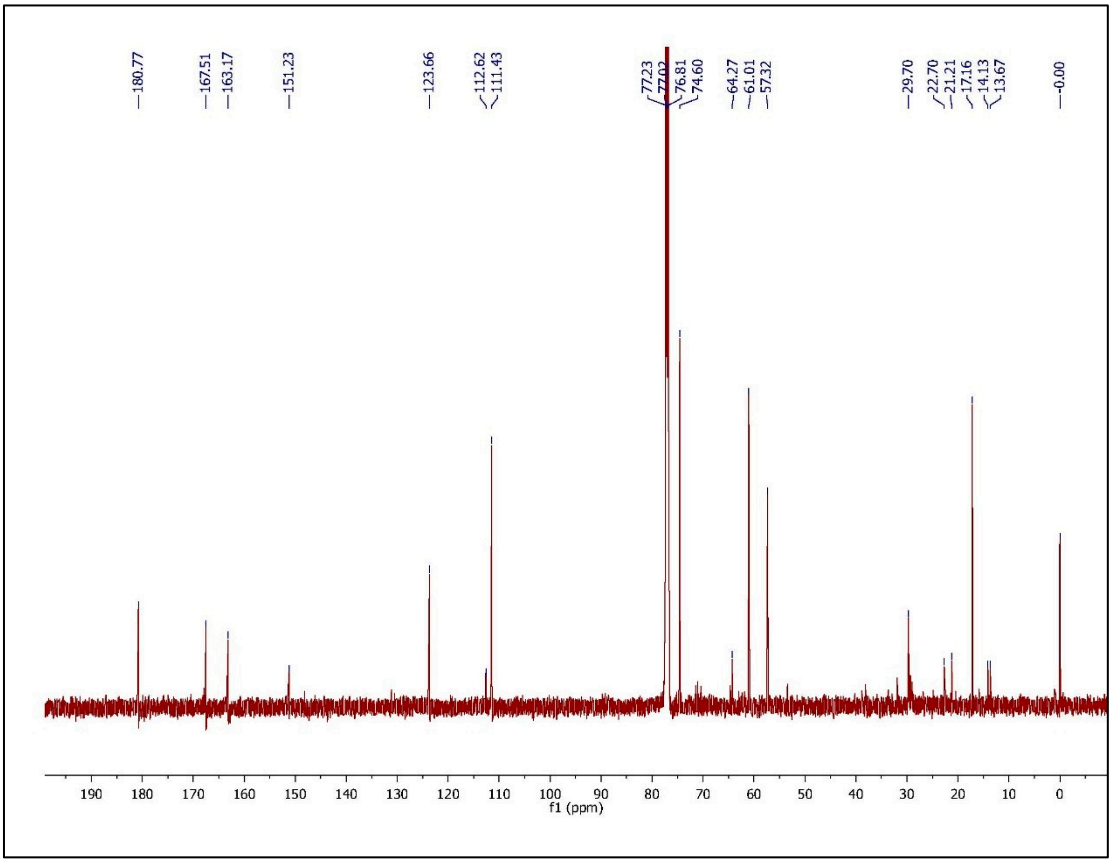

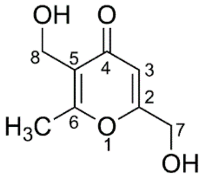

Table S2. ^{1}H and ^{13}C NMR spectroscopic data of herierin III.

Position	^{1}H (ppm)[1]	^{13}C (ppm)[2]
2		167.5
3	6.40 s	111.4
4		180.8
5		123.7
6		163.2
7	4.46 s	61.0
8	4.52 s	57.3
6-CH_3	2.37 s	17.2

Assignments based on COSY, HSQC and HMBC. Recorded at 600 MHz in MeOH-*d*4.[1]

Recorded at 150 MHz in MeOH-d4.[2]

Figure S5. ^{1}H NMR (MeOH-d_4, 600 MHz) spectrum of herierin IV.

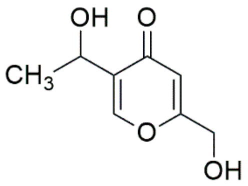

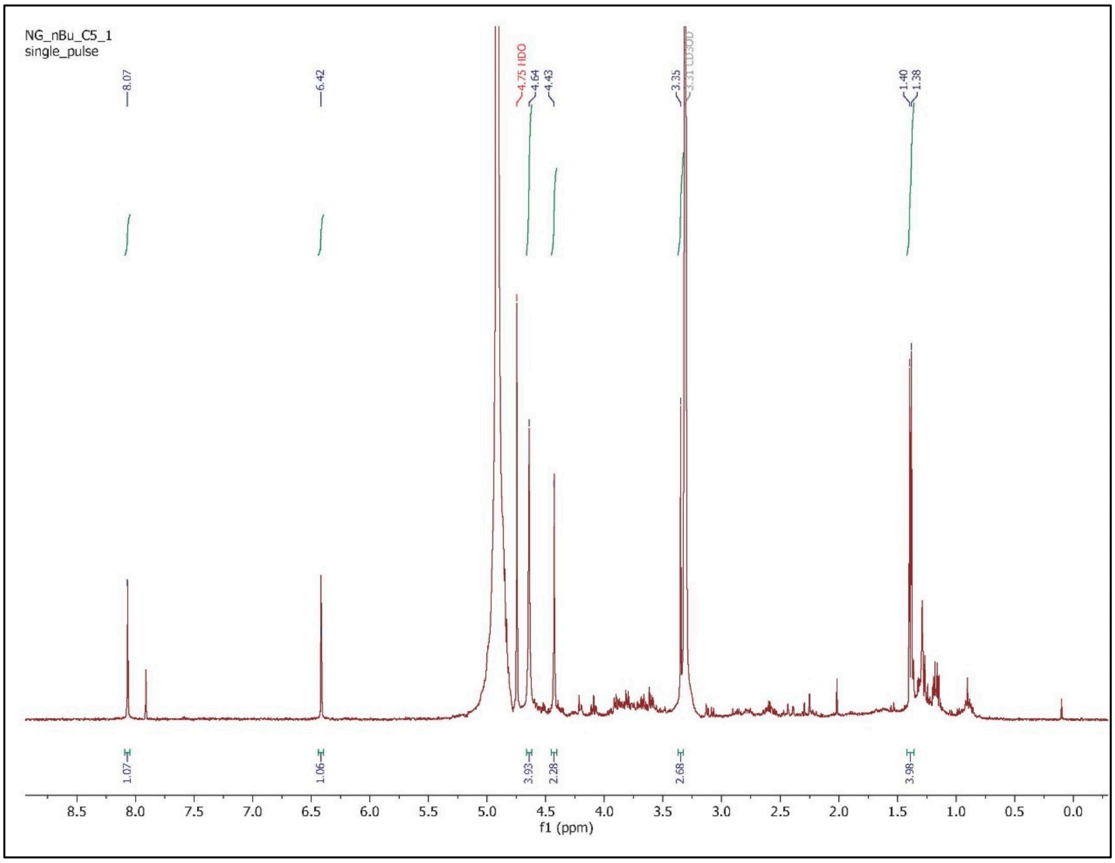

Figure S6. ^{13}C NMR (MeOH-d_4, 150 MHz) spectrum of herierin IV.

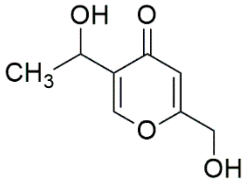

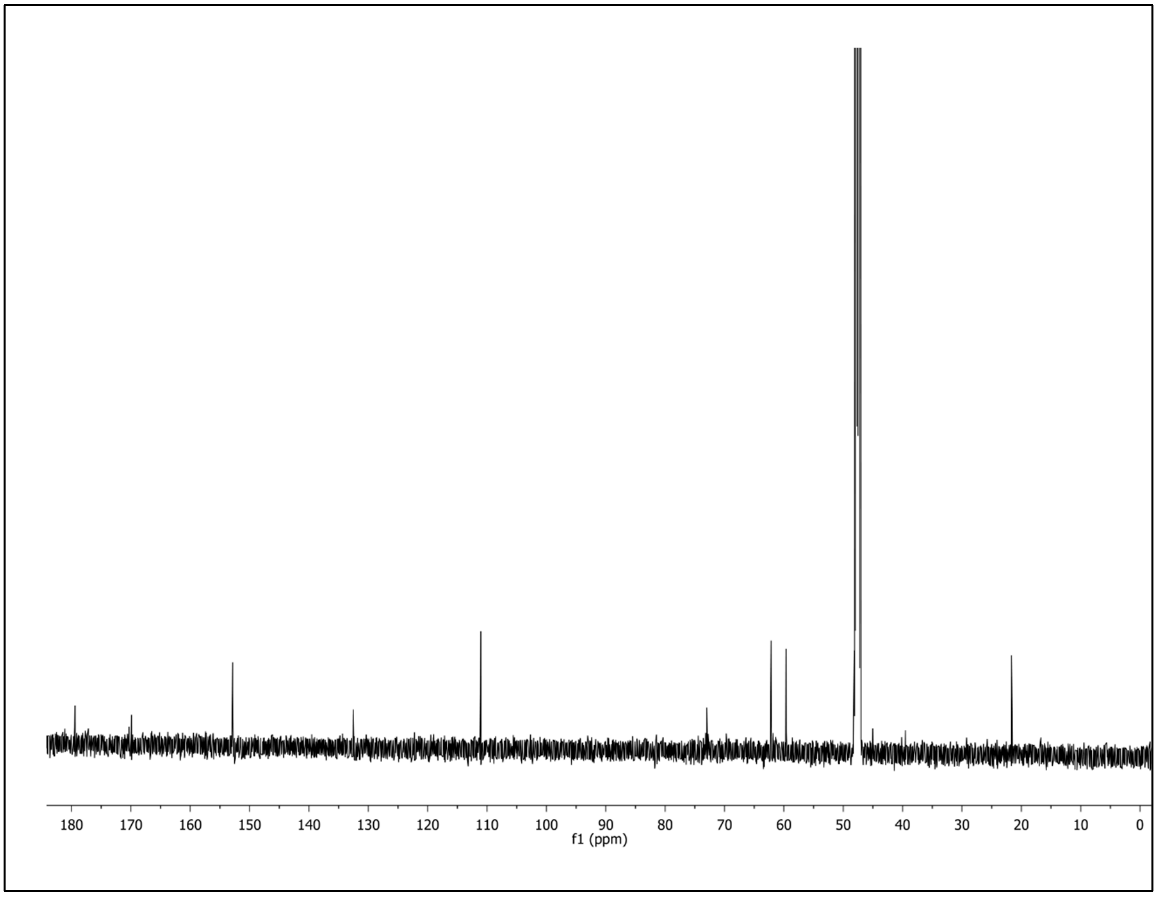

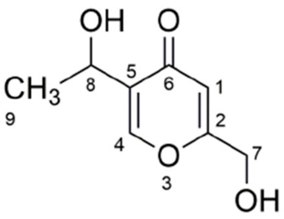

Table S3. ^{1}H and ^{13}C NMR spectroscopic data of herierin IV.

**Position**	**^{1}H (ppm)[1]**	**^{13}C (ppm)[2]**
1	6.42 s	112.5
2		171.3
4	8.07 s	171.7
5		134.0
6		180.8
7	4.64 s	61.0
8	4.43 s	63.6
9-CH_3	1.38 d	23.0

Assignments based on COSY, HSQC and HMBC. Recorded at 600 MHz in MeOH-*d*4.[1]

Recorded at 150 MHz in MeOH-d4.[2]

Figure S7. Effects of 3-AP and *H.E.* treatment on animal body weight and motor coordination and balance. (A) Body weight changes during the experimental period. (B) Pre-treatment relative latency to fall (C) Relative percentage deficits of latency to fall of accelerated rotarod test. (D) Relative latency to fall of rod test. Indicators: *P-values ≤ 0.05, **P-values ≤ 0.01.

Figure S7

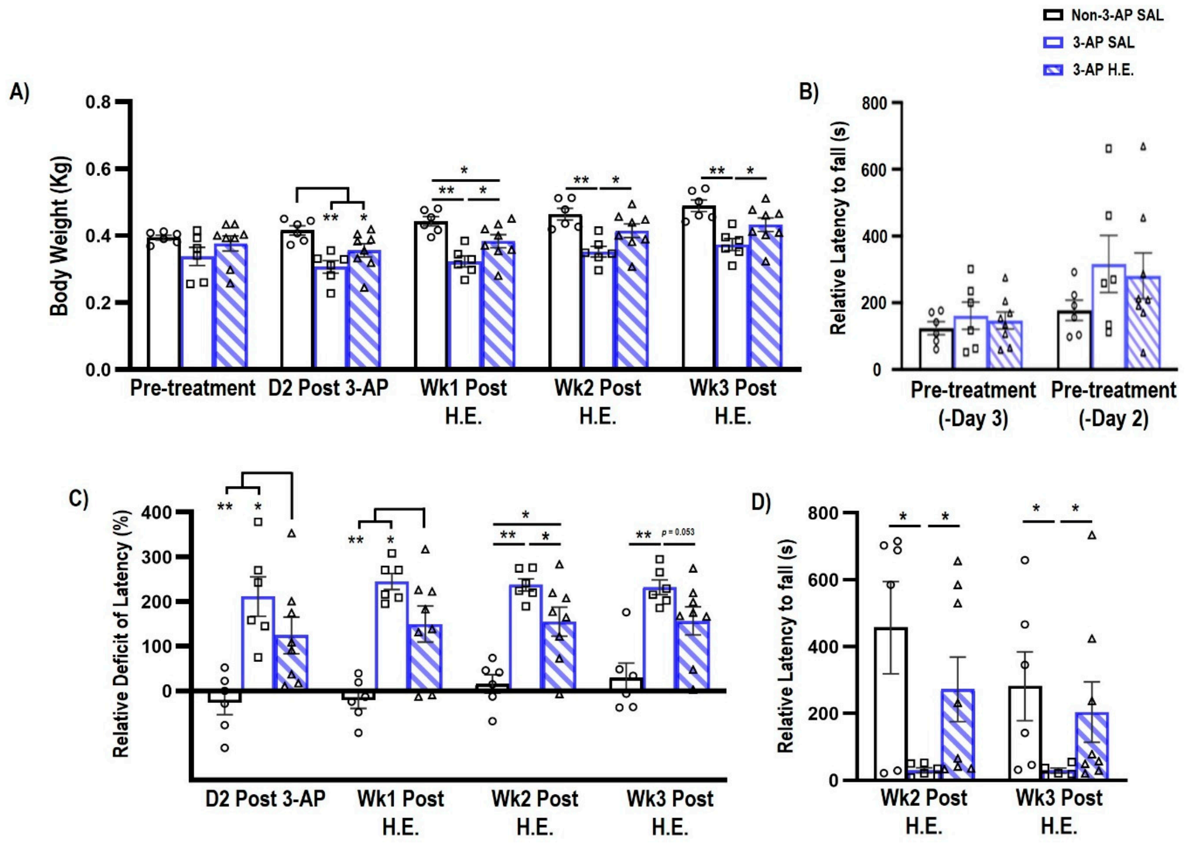

Supplement: Supplementary file 1 [file ijms-24-06089-s001.zip › ijms-2285884-supplementary.pdf]
